# Left bundle branch pacing in double-chambered left ventricle and perimembranous ventricular septal aneurysm: a case report

**DOI:** 10.1093/ehjcr/ytag681

**Published:** 2026-09-12

**Authors:** Kristina Brečko, Anja Zupan Mežnar, Nejc Pavšič, Mojca Bervar, David Žižek

**Affiliations:** Faculty of Medicine, University of Ljubljana, Vrazov trg 2, 1000 Ljubljana, Slovenia; Faculty of Medicine, University of Ljubljana, Vrazov trg 2, 1000 Ljubljana, Slovenia; Department of Cardiology, University Medical Centre Ljubljana, Zaloška 2, 1000 Ljubljana, Slovenia; Department of Cardiology, University Medical Centre Ljubljana, Zaloška 2, 1000 Ljubljana, Slovenia; Department of Cardiology, University Medical Centre Ljubljana, Zaloška 2, 1000 Ljubljana, Slovenia; Faculty of Medicine, University of Ljubljana, Vrazov trg 2, 1000 Ljubljana, Slovenia; Department of Cardiology, University Medical Centre Ljubljana, Zaloška 2, 1000 Ljubljana, Slovenia

**Keywords:** Double-chambered left ventricle, Perimembranous septal aneurysm, Congenital heart disease, Complete heart block, Conduction system pacing, Case report

## Abstract

**Background:**

Double-chambered left ventricle and perimembranous ventricular septal aneurysm are rare congenital heart abnormalities that often remain undetected until adulthood. Both abnormalities can be associated with conduction disorders requiring cardiac pacing. To our knowledge, this is the first case report of a patient with both a double-chambered left ventricle and a perimembranous ventricular septal aneurysm, undergoing left bundle branch area pacing (LBBAP) due to complete atrioventricular (AV) block.

**Case summary:**

A 31-year-old man with complete AV block was admitted to our centre, where diagnostic evaluation revealed a complex congenital heart defect: a large perimembranous septal aneurysm and a double-chambered left ventricle. Due to high predicted pacing burden, conduction system pacing with LBBAP was attempted. In addition to the standard technique, transoesophageal echocardiography (TEE) was used intraoperatively to facilitate safe positioning of the LBBAP lead. Finally, engagement of the conduction system was confirmed and preserved at the 1-year follow-up, with stable pacing parameters.

**Discussion:**

Conduction system pacing in congenital heart defects can be technically challenging due to complex anatomy. This case highlights LBBAP as a feasible physiological pacing option, even in complex and rare congenital heart defects. To minimize procedural risk, careful preprocedural multimodality imaging and intraoperative utilization of TEE were essential for optimal lead positioning in the setting of abnormal septal structures.

Learning points

- Left bundle branch area pacing is feasible in double-chambered left ventricle and a perimembranous ventricular septal aneurysm.
- Adjunctive intraoperative transoesophageal echocardiography can facilitate conduction system pacing in patients with rare congenital heart disease.

## Introduction

A double-chambered left ventricle (DCLV) is a rare congenital heart defect characterized by the division of the left ventricle into two chambers due to an abnormal septum or muscle band. It can often be asymptomatic and discovered incidentally in adulthood.^1^ In comparison to a double-chambered right ventricle, which accounts for 0.5%–2.0% of all congenital heart diseases (CHD), DCLV is even rarer with only few cases reported, mostly illustrating the different variants or imaging characteristics of the abnormality.^2,3^ To our knowledge, this is the first case report of left bundle branch area pacing (LBBAP) in a patient with DCLV and perimembranous ventricular septal aneurysm (PMSA).

## Summary figure

**Figure:**
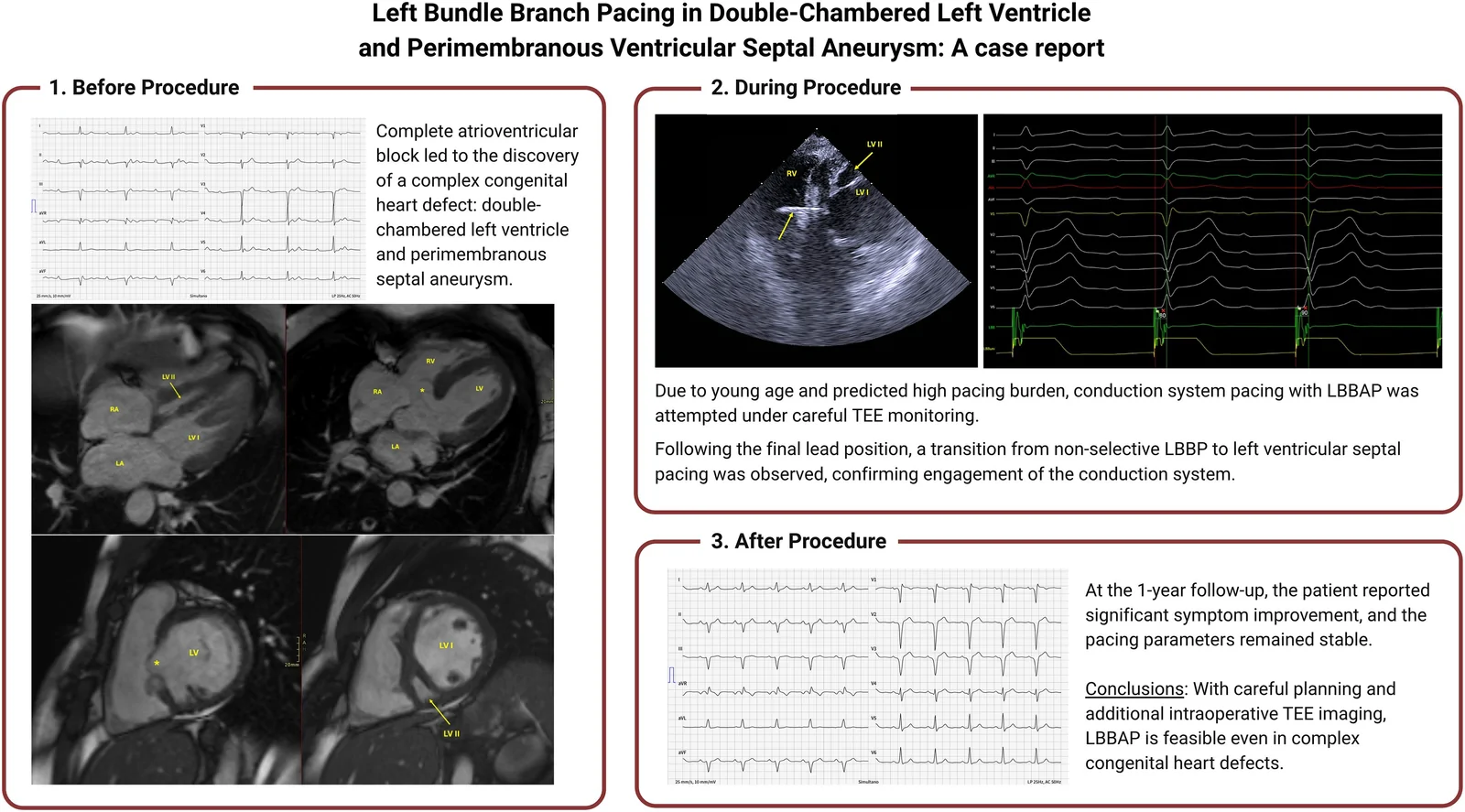


## Case presentation

**Figure 1:**
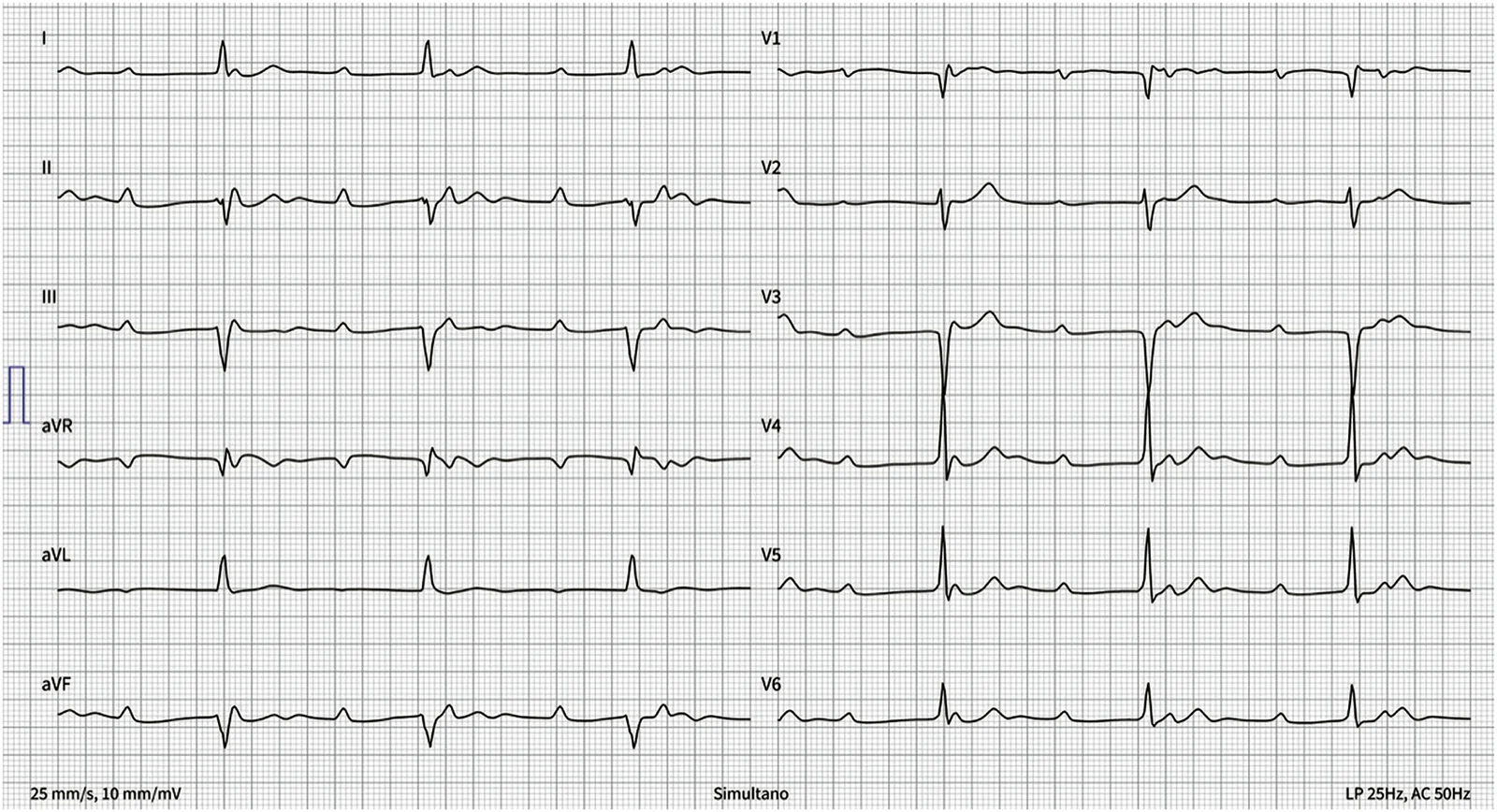
Baseline 12-lead electrocardiogram. Tracing is showing third degree atrioventricular block with narrow QRS complexes and an escape rhythm of 42 bpm.

**Figure 2:**
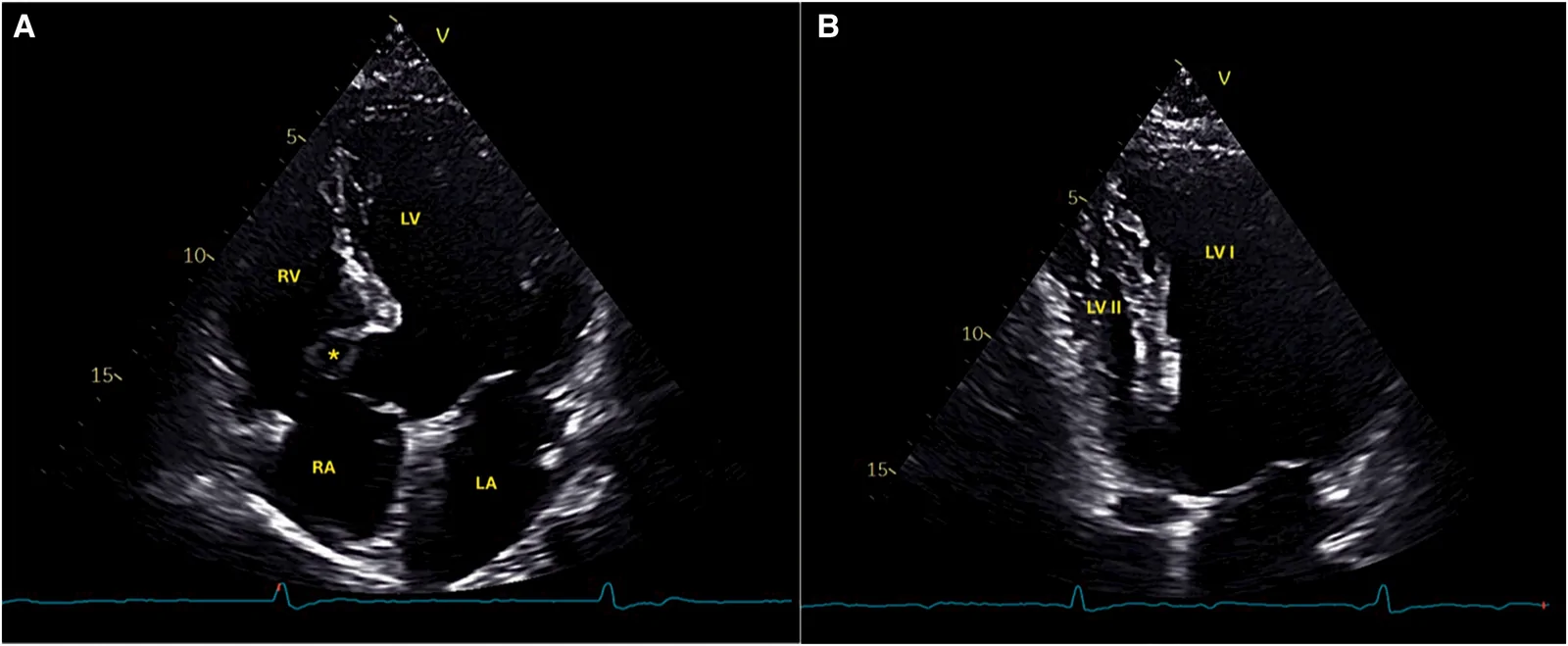
Transthoracic echocardiographic images showing a perimembranous interventricular septal aneurysm (marked with asterisk) and a left ventricular accessory chamber (LV II). (*A*) Two-dimensional TTE apical four-chamber view. (*B*) Apical two-chamber view. LA, left atrium; LV/LV I, left ventricle/primary chamber of the left ventricle; LV II, accessory chamber of the left ventricle; RA, right atrium; RV, right ventricle.

**Figure 3:**
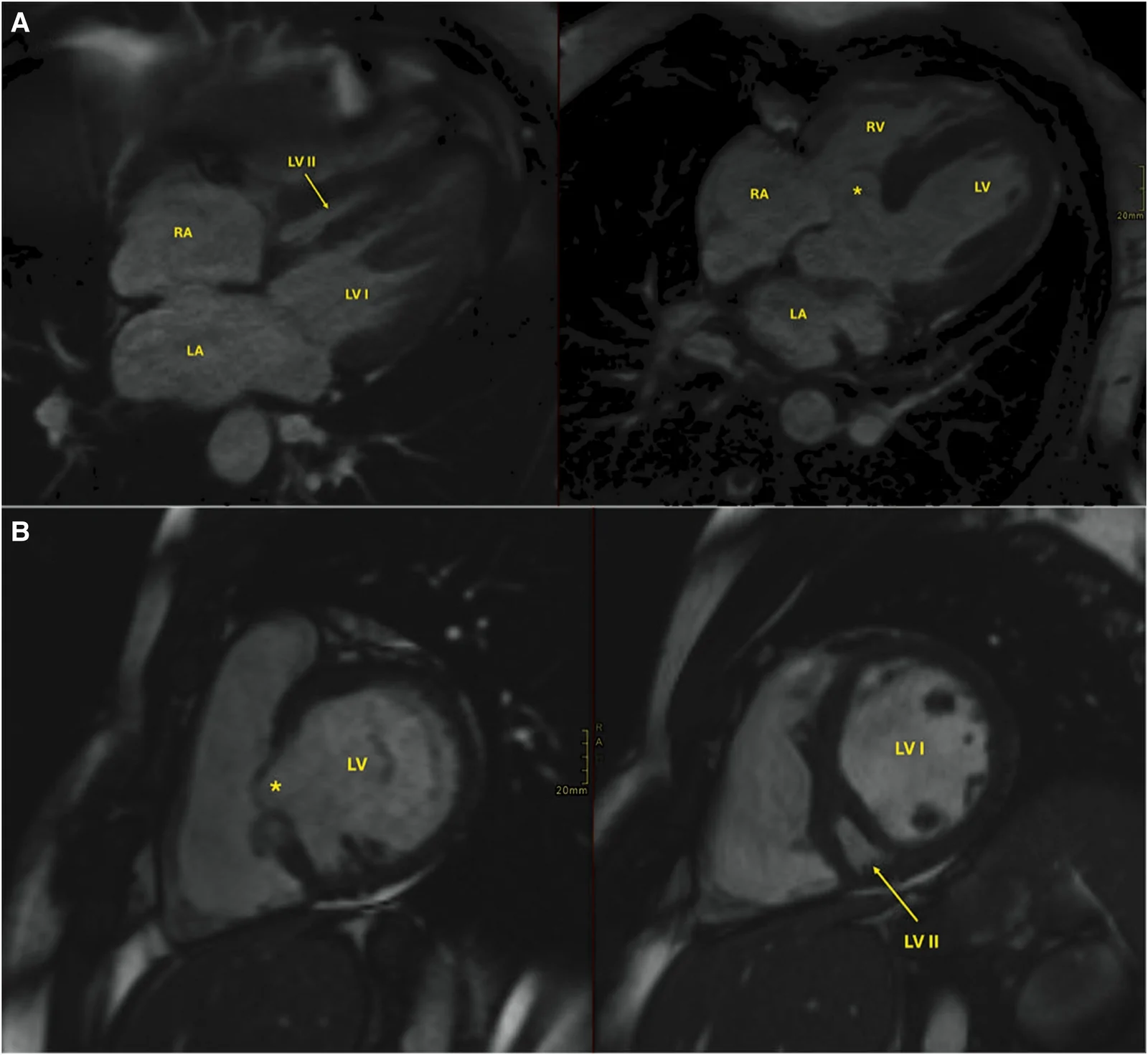
Cine cardiac magnetic resonance images demonstrating a perimembranous interventricular septal aneurysm (marked with asterisk) and a left ventricular accessory chamber (LV II). (*A*) Apical four-chamber view in systole. (*B*) Short-axis two-chamber view. LA, left atrium; LV/LV I, left ventricle/primary chamber of the left ventricle; LV II, accessory chamber of the left ventricle; RA, right atrium; RV, right ventricle.

**Figure 4:**
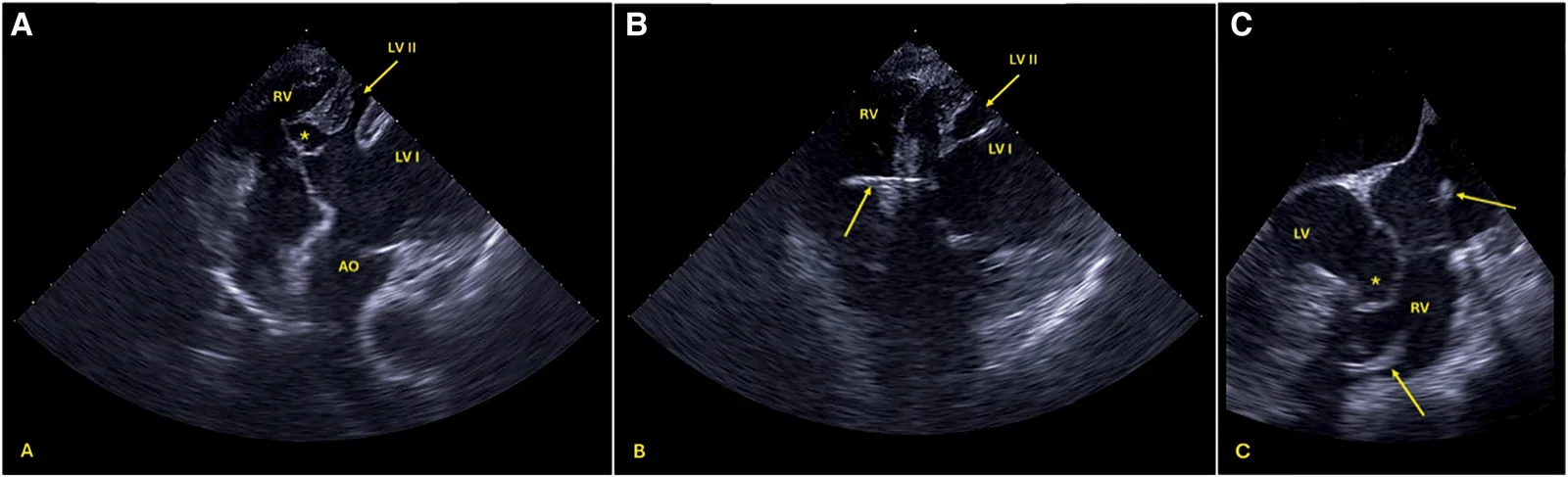
Transoesophageal echocardiographic (TEE) images during left bundle branch pacing lead implantation. (*A*) Two-dimensional TEE in deep *trans*-gastric long-axis (DTG LAX) view demonstrating anatomical correlation between the perimembranous interventricular septal aneurysm (marked with asterisk) and the accessory chamber in the left ventricle (LV II). (*B*) Two-dimensional TEE in DTG LAX demonstrating lead (indicated as arrow) deployment in the septum in correlation with the accessory chamber (LV II). (*C*) Two-dimensional TEE in mid-oesophageal view (208°) demonstrating the relationship between the cathete or the lead (arrow), and the aneurysm (marked with asterisk). AO, aortic valve; LV/LV I, left ventricle/primary chamber of the left ventricle; LV II, accessory chamber of the left ventricle; RV, right ventricle.

**Figure 5:**
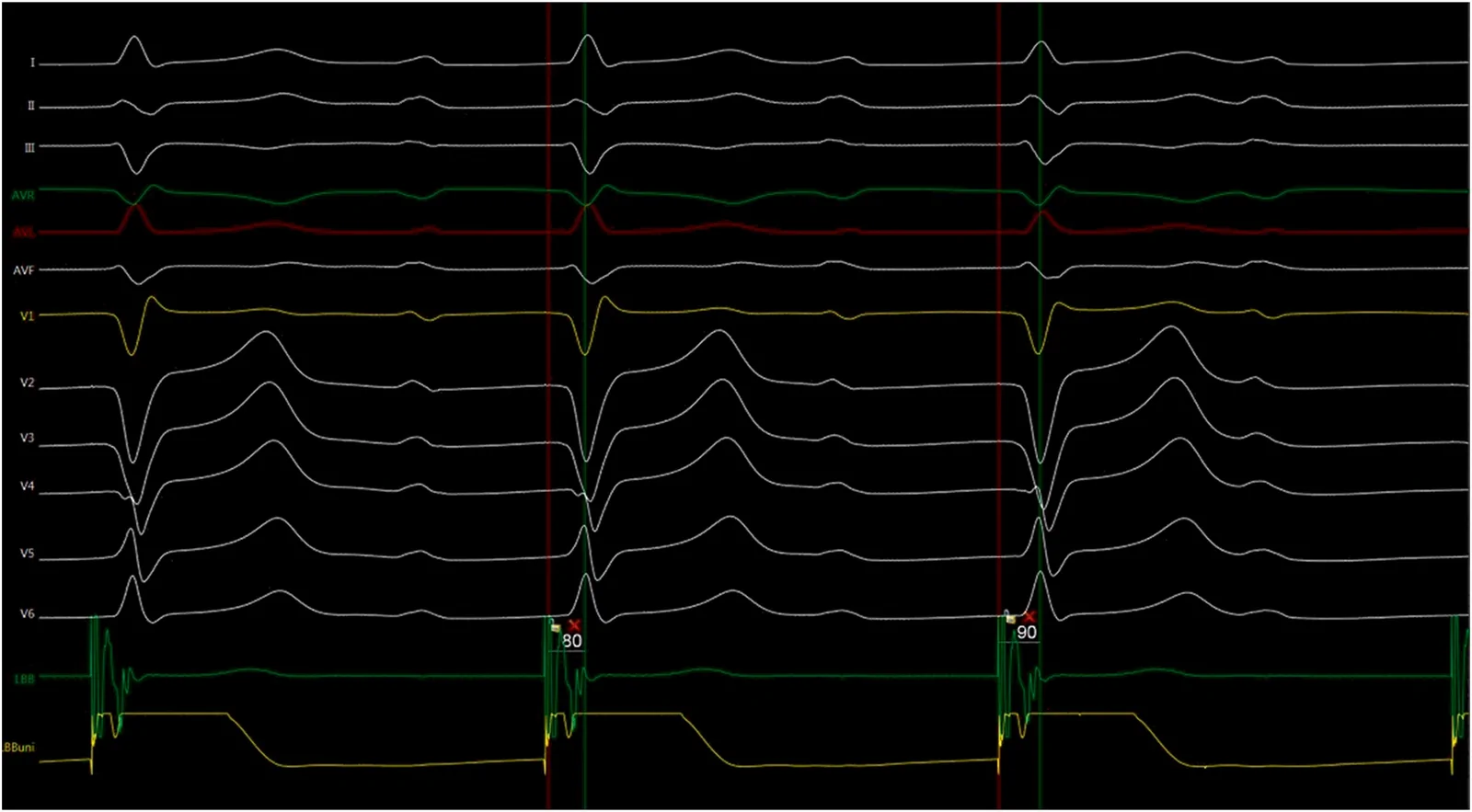
Intraoperative 12-lead electrocardiogram and filtered and unfiltered intracardiac electrograms during threshold test, QRS transition in lead V1 can be observed with prolongation of R wave peak time in lead V6 (from 80 to 90 ms)—indicating transition from non-selective left bundle branch pacing to left ventricular septal pacing; unfiltered intracardiac electrogram (yellow) shows current of injury typical for left endocardial lead placement.

A 31-year-old man presented to his general practitioner for his first routine check-up, reporting exertional dyspnoea but denying syncope or presyncope. A 12-lead electrocardiogram revealed a complete atrioventricular block with narrow QRS complexes and an escape rhythm of 42 bpm (*Figure 1*), prompting referral to our centre. Family history was significant for cardiovascular disease: His father and paternal uncle both had dilated cardiomyopathy with reduced ejection fraction and received an implantable cardioverter–defibrillator and a cardiac resynchronization therapy defibrillator (CRT-D), respectively. Physical examination revealed no abnormalities. Transthoracic echocardiography (TTE) revealed a large PMSA, without ventricular septal defect, and a significantly enlarged left ventricle (end-diastolic volume index: 111 ml/m^2^) with a normal left ventricular ejection fraction (54%) (*Figure 2*). Cardiac magnetic resonance imaging additionally showed a division of muscular interventricular septum, measuring 10 mm and communicating basally with the left ventricle, consistent with a DCLV (*Figure 3*). The left ventricle was enlarged, without evidence of focal or diffuse myocardial fibrosis. Due to the patient's young age and predicted high pacing burden, conduction system pacing (CSP) with LBBAP was proposed. Device implantation was performed under general anaesthesia with additional transoesophageal echocardiography (TEE) guidance to avoid the PMSA and the divided part of the septum. Specifically, a standard mid-oesophageal four-chamber view (0°–30°) was used to visualize the tricuspid valve and guide the lead past the interatrial septum, aneurysm, and interventricular septum, while a sub-tricuspid *trans*-gastric view confirmed lead deployment within the muscular interventricular septum. Due to asystole risk during the procedure, atrial lead was first placed in the apex of the right ventricle as a back-up lead. For LBBAP lead placement, C315HIS delivery catheter (Medtronic, MN, USA) and a SelectSecure 3830 lead (model 3830, Medtronic, MN, USA) were used. Under fluoroscopy (right anterior oblique view 20°) and TEE guidance, the delivery catheter was carefully advanced through the tricuspid valve and positioned below the PMSA (*Figure 4*). Once fluoroscopic and echocardiographic imaging had confirmed that the delivery sheath was positioned in the undivided interventricular septum, the pacing lead was advanced through the sheath. The initial pacing site was further optimized with counterclockwise torque until pacing resulted in a W-shaped QRS complex in lead V1, a predominantly positive QRS complex in lead II, and a predominantly negative QRS complex in lead III, consistent with mid-septal position. The lead was then gradually screwed deep into the septum until unipolar pacing at the site showed a right bundle branch block QRS morphology with a notch in the nadir of the QS complex. With QRS transition from non-selective LBBP to left ventricular septal pacing (LVSP) during the threshold test, an interval from stimulus to peak of R wave in lead V6 of 80 ms, an isoelectric interval on intracardiac electrogram, and a V6–V1 interpeak interval of 40 ms, conduction system engagement was confirmed (*Figure 5*). Final LBBAP lead parameters were excellent (transition threshold at 1.5 V and LVS capture threshold at 0.5 V at 0.5 ms; pacing impedance: 700 Ω; R-wave amplitude: 11.0 mV). After successful sheath removal, atrial lead (CapSureFix Novus 4076-52 cm, Medtronic) was placed in the right atrial appendage (capture threshold at 0.5 V at 0.4 ms; pacing impedance: 513 Ω; P-wave amplitude: 3.0 mV).

The total procedure time was 72 min, with 11 min of fluoroscopy. There were no periprocedural complications. The device was programmed to DDD mode with a lower rate of 50 bpm and upper tracking of 150 bpm and the patient was discharged the following day.

At the 1-year follow-up, the patient reported considerable improvement in quality of life and increased exercise tolerance. In addition, TTE demonstrated a reduction in left ventricular size (end-diastolic volume index: 72 ml/m^2^) and preserved left ventricular ejection fraction (LVEF 56%). Atrial and ventricular lead parameters remained stable.

## Discussion

To our knowledge, we present the first case report of CSP with LBBAP in a patient with a DCLV and PMSA. PMSA has been reported in 0.3% of patients with CHD and can be either idiopathic or related to spontaneous closure of a ventricular septal defect, often found incidentally.^4^ The coexistence of DCLV and PMSA represents a remarkable rarity, and while both malformations are commonly associated with other cardiac abnormalities,^5,6^ we found no previous reports describing their combined occurrence in a single patient. The patient's family history could suggest a possible familial cluster, similarly to what is described by Zhang *et al.*^3^; however, because advanced imaging was never performed in the relatives, further information regarding their exact cardiac morphology could not be obtained.

The aetiology of complete AV block in our patient is unclear. Advanced conduction disturbances have previously been reported in patients with PMSA, while, to our knowledge, no such cases have been described in patients with DCLV. Clinically, conduction abnormalities reported in patients with PMSA range from bundle branch block and first-degree atrioventricular block to complete atrioventricular block. Although pacemaker implantation due to complete AV block has been reported in patients with PMSA,^7,8^ experience with LBBAP has not previously been described.

Compared with conventional RV pacing, CSP provides a more physiological ventricular activation pattern and may better preserve long-term ventricular function, particularly in younger patients with CHD and high pacing burden, who are more susceptible to pacing-induced cardiomyopathy.^9^ Despite the technical challenges posed by complex congenital anatomy, CSP is associated with high procedural success. However, long-term lead-related complications, including microdislodgement, rising capture thresholds, and loss of conduction system capture, remain potential concerns, particularly in patients with abnormal septal anatomy.^10^ These complications appear to be less frequent with LBBAP than with His bundle pacing.^10,11^

In this case, transseptal implantation of the LBBAP lead was particularly challenging because the presence of a PMSA distorted the normal septal anatomy and increased the risk of inadvertent lead advancement into or through the aneurysmal tissue. Reliance on conventional fluoroscopic landmarks and paced QRS morphology alone may have been insufficient to accurately identify the optimal implantation site, potentially increasing the risk of aneurysm perforation, lead instability, or placement within the accessory paraseptal left ventricular chamber. Consequently, meticulous preprocedural multimodality imaging was essential to define the individual septal anatomy and guide procedural planning. During implantation, TEE guidance complemented fluoroscopy and intracardiac electrogram assessment by confirming perpendicular sheath orientation, identifying the undivided muscular septum as the target for lead deployment, and continuously monitoring lead advancement to avoid the aneurysm and accessory chamber. This integrated imaging approach enabled safe transseptal lead implantation while minimizing the risk of procedural complications. In patients with abnormal septal anatomy, the combined use of TEE, fluoroscopy, and electrogram guidance should therefore be considered to facilitate accurate and safe LBBAP lead placement.

Although TEE was crucial in the present case, intracardiac echocardiography (ICE) represents a potential alternative. ICE provides real-time imaging without oesophageal intubation and allows the implanting physician to directly control image acquisition, often under conscious sedation.^12^ However, it requires additional venous access and a dedicated imaging catheter, increasing procedural cost, while conventional ICE catheters have more limited multiplanar and three-dimensional imaging capabilities than TEE.^13^ Therefore, the choice between TEE and ICE should be guided by patient characteristics, anatomical complexity, and local expertise.

In this case, mid-septal (non-basal) initial catheter alignment was used for LBBAP to avoid areas with anatomical abnormalities. After confirming non-selective engagement of the posterior fascicle, it was noted that lower (mid-apical) positioning might have hindered effective conduction system capture. However, it is important to emphasize that in complex cases with anatomical variations, prioritizing a stable lead position with acceptable pacing parameters is essential. Even though LVS pacing may be considered inferior to true conduction system capture, it still produces better outcomes compared to conventional RV pacing.^14^

In conclusion, management of bradycardia in adults with CHD requires careful preoperative planning and detailed knowledge of the underlying anatomy.^15^ CSP with LBBAP offers a feasible physiological pacing option, even in complex and rare CHD cases. Intraoperative TEE can complement fluoroscopy and electrogram guidance by assisting with lead positioning and potentially minimizing procedural complications. Nevertheless, long-term outcome data on LBBAP in rare congenital anatomical variants remain limited and warrant further investigation.

## Lead author biography


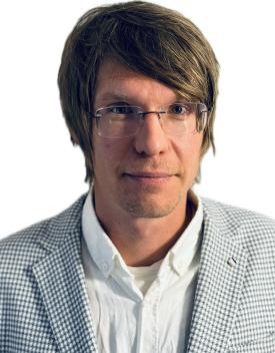


Head of Laboratory for Cardiac Stimulation and Resynchronization, and leads research programme in implantable cardiac devices.

## Supplementary Material

ytag681_Supplementary_Data

## Data Availability

Data used in this paper are available upon specific request to the corresponding author.
